# Effect of a rapid fat loss protocol on body composition and weight regain in midlife females: a case series study preliminary analysis

**DOI:** 10.1080/15502783.2025.2550190

**Published:** 2025-08-26

**Authors:** Landon Shannahan, Mateus Muniz, Valeria V. Silva Ramirez, John Holtje, Jakob Paz, Gretchen Shelton, Bill I. Campbell

**Affiliations:** University of South Florida, Performance & Physique Enhancement Lab, Exercise Science & Kinesiology Program, Tampa, FL, USA

**Keywords:** Weight loss, sustainability, diet, women

## Abstract

**Background:**

Successful dieting is not solely defined by weight loss, but by the composition and long-term maintenance of that loss. Weight regain is common post-diet, with only ~ 25% of individuals maintaining weight loss. Few studies have been done on the effectiveness and sustainability of rapid weight loss protocols. Our case-series study aims to assess the efficacy of a Rapid Fat Loss protocol (RFL) in midlife females on fat loss, fat-free mass preservation, and weight regain.

**Methods:**

This preliminary descriptive analysis includes seven resistance-trained females (55 ± 6 years). Following a two-week weight maintenance phase, participants completed a four-day RFL protocol consisting of 1.6 g protein/kg bodyweight, trace carbohydrates and fats, and six hours of walking per day. They returned to maintenance intake for three days, then transitioned to 30 days of ad libitum eating. Body composition was assessed at baseline, post-RFL (Day 7), and follow-up (Day 30) via the Siri 3-compartment model. Reported values reflect means ± SD. Three participants are pending follow-up.

**Results:**

After 7 days, participants lost − 1.73 ± 1.22 kgs, driven by reductions in fat mass (FM: −1.53 ± 1.03 kgs) with minimal FFM loss (−0.2 ± 0.68 kgs) and a − 1.7 ± 1.25% decrease in percent body fat (PBF). After 30 days of ad-libitum intake, net weight change was − 1.95 ± 1.64 kgs, net FM decreased further (−2.23 ± 1.42 kgs), net FFM increased (+0.28 ± 0.38 kg), and net PBF further decreased (−2.67 ± 1.41%). Between Day 8–30, weight remained stable (0.05±.62 kgs) with continued FM loss (−0.45 ± 0.9) and an increase in FFM = (0.53 ± 0.61).

**Conclusions:**

This 7-day RFL protocol appears to elicit favorable, sustainable body composition outcomes in midlife females with no observed short-term weight regain.

